# Alterations of DNA methylation and expression of genes related to thyroid hormone metabolism in colon epithelium of obese patients

**DOI:** 10.1186/s12920-022-01387-6

**Published:** 2022-11-01

**Authors:** Ghazaleh Shimi, Katayoun Pourvali, Arman Ghorbani, Sajad Nooshin, Shohreh Zare Karizi, Reza Iranirad, Hamid Zand

**Affiliations:** 1 Department of Cellular and Molecular Nutrition, Faculty of Nutrition Science and Food Technology, National Nutrition and Food Technology Research Institute, Shahid Beheshti University of Medical Sciences, Tehran, 1981619573 Iran; 2 MSc Molecular Genetics, Islamic Azad University, Pishva-Varamin, Iran; 3 Department of Genetics, Islamic Azad University, Varamin-Pishva Branch, Varamin, Iran; 4 Sasan Alborz Biomedical Research Center, Masoud Gastroenterology and Hepatology Clinic, Tehran, 14117-13135 Iran

**Keywords:** DNA methylation, Obesity, *THRB*, *DIO3*, Colorectal cancer

## Abstract

**Background:**

Colorectal cancer is common among obese individuals. The purpose of the current study was to determine changes in DNA methylation status and mRNA expression of thyroid hormone receptor beta (*THRB*), as a tumor suppressor, and thyroid hormone inactivating enzyme, type 3 deiodinase (*DIO3*) genes, in human epithelial colon tissues of healthy obese individuals.

**Methods:**

Colon biopsies were analyzed by methylation sensitive-high resolution melting (MS-HRM) to investigate promoter methylation of *DIO3* and *THRB*, and by quantitative real-time polymerase chain reaction to assay expression of *DIO3* and *THRB* mRNA on eighteen obese and twenty-one normal-weight healthy men.

**Results:**

There was no significant difference in mean methylation levels at the *THRB* promoter region between the two groups. Nevertheless, obesity decreased *THRB* expression levels, significantly (*P* < 0.05; fold change: 0.19). Furthermore, obesity attenuated DNA methylation (*P* < 0.001) and enhanced mRNA expression of *DIO3* (*P* < 0.05; fold change: 3).

**Conclusions:**

Our findings suggest that obesity may alter expression of *THRB* and *DIO3* genes through epigenetic mechanism. Alterations of *THRB* and *DIO3* expressions may predispose colon epithelium of obese patients to neoplastic transformation.

## Background

Obesity is associated with an increased risk of some types of cancer including, colorectal, breast, endometrial, esophageal, pancreatic, and kidney [1]. Despite a recognized role of obesity in increasing oncogenesis, the mechanisms by which obesity regulates tumor initiation and growth are not yet clear. Among the most important proposed mechanisms linking obesity and cancer are obesity-induced low-grade inflammation and obesity-associated dysfunction of insulin/IGF1 signaling [2]. According to a study conducted by Homayounfar et al. (2015), p53 tumor suppressor, as a main guardian of cells against neoplastic transformation, is upregulated in intestine and some other tissues of obese rats [3]. Therefore, predisposition of obese patients to neoplastic alterations might result from the loss of other tumor preventive signaling through genetic or epigenetic inactivation.

The link between thyroid hormones (THs) and cancer was proposed more than a decade ago. Most epidemiological evidence shows that hypothyroidism prevents tumor initiation and growth, however some other studies provide opposite results [4]. THs exert their effects through genomic and non-genomic pathways. Genomic actions of THs take place through thyroid hormone receptors (TRs) including TRα and TRβ and their various isoforms [5]. Non-genomic actions of THs are exerted via signaling originated from membrane receptors, integrin avβ3, or through interaction with cytoplasmic kinases, such as phosphoinositide 3-kinase (PI3K). Plasma membrane integrin avβ3 is upregulated in cancer cells and promotes its intracellular signaling through PI3K and mitogen-activated protein kinase (MAPK)/ERK1/2 pathways [6–8].

According to several lines of evidence, pro-cancerous effects of THs are exerted via either TRα or integrin avβ3 by promoting angiogenesis, proliferation, apoptosis suppression, evasion from immune surveillance, and metabolic reprogramming [9]. Convincing evidence suggests that TRβ is a tumor suppressor in several types of cancer, including thyroid, breast, colorectal and other solid tumors [10]. Downregulation of TRβ is the characteristic of many cancers [10]. TRβ1 is able to suppress proliferation and migration in human colorectal cancer cells, and has been proposed to be a target for treating CRC [11].

Expression of type 3 deiodinase, as an inactivating thyroid hormone enzyme, is negligible in adult tissues; however, its re-expression was shown in some pathological conditions such as tissue regeneration after injury, inflammation and cancers such as breast, endometrium carcinoma, basal cell carcinoma, and colon [12]. In most colon tumors, β-catenin/T-cell factor (TCF) complex is activated. Increased DIO3 levels, as a direct downstream target of the Wnt/β-catenin pathway, reduce intracellular T3 and E-cadherin, promote cellular proliferation, and prevent differentiation [13].

Our present research studied the alterations of *THRB* and *DIO3* genes at epigenetic and mRNA expression levels in colonic epithelium specimens of obese individuals. This can provide evidence for obesity-related changes in THs metabolism that predispose colon tissues to cancer development.

## Methods

### Participants

The participants in this case–control study were consecutively selected from Modarres hospital and Masoud clinic (18 obese and 21 normal-weight men), between December 2018 and April 2019 in Tehran, Iran. They were enrolled from the ambulatory endoscopy clinic of the hospital and colonoscopy unit of the clinic. Inclusion criteria were: individuals between the ages of 25 and 60, BMI ≥ 30 kg/m^2^ for obese men as case and BMI between 18 and 25 kg/m^2^ for normal-weight men as control, with no acute or chronic illness and taking no medication. Individuals with a personal history of colonic neoplasia, colitis, polyposis, previous colon resection, and CRC or different types of cancer were excluded. DNA methylation and mRNA expression analyses were conducted on all participants.

All men were on a low-fiber diet for 3 days before the colonoscopy. On the day of colonoscopy, they were fasted. Standard optical colonoscopy was carried out under the direct supervision of attending gastroenterologists. All colonic biopsies were taken from mid-rectum by using jumbo pinch forceps. Finally, the biopsies were rapidly snap-frozen in liquid nitrogen, transported to laboratory, and stored at − 80 °C.

Weight and height of participants were measured with standard protocols, and body mass index (BMI) was calculated as weight in kilograms divided by height in square meter. Waist: hip ratio (WHR), waist circumference (WC), and hip circumference were also measured using a non-stretchable tape. General information was also recorded from the participants. This study was conducted according to the criteria set by the declaration of Helsinki [14], and written informed consent forms were obtained from all participants before recruitment.

### DNA extraction and sodium bisulfite treatment

DNA was extracted from the colon tissue samples using the animal DNA Mini-Preps Kit (Bio Basic Inc.), according to the manufacturer’s protocol. Extracted DNA samples were treated by sodium bisulfite conversion, using an EZ DNA Methylation Gold kit (Zymo Research). The treated DNA samples were rapidly stored at − 80 °C.

### Methylation sensitive-high resolution melting

MS-HRM was used to assess the methylation status of *DIO3* and *THRB* genes. Primers for MS-HRM assay were designed according to HRM primer design principles. PCR was carried out in a 20 μL total volume containing 1 µL bisulfite modified template, 4 µL of 5 × Hot FIREPOL Eva Green HRM Mix-Rox Kit (Solis BioDyne), 14 µL double-distilled water, and 6 pmol/µL of forward and reverse primers (1 µL). DNA melting with high resolution was a three-stage process. The first stage included initial denaturation at 95 °C for 15 min. The second stage consisted of three steps in 40 cycles: 95 °C for 15 s, appropriate annealing temperature for each primer set (Table 1) for 20 s, 72 °C for 30 s (extension). The third stage (melting curve continuous stage) was performed as follows: 95 °C for 15 s, 60 °C for 1 min, followed by HRM step ramping from 60 to 95 °C, rising at 0.3 °C per second (StepOnePlus, Applied Biosystems, Paisley, UK).

**Table 1:** Methylation-sensitive high-resolution melting primers, designed for promoter regions, and their amplicon information

Gene name	Sequence (5′ → 3′)	Ta˚C	Accession number	Number of CpG-sites/amplicon length
*DIO3*	F: GAG GGT ATT GTA GTA AGG TGT ATT R: AAA ACC CAA CCC ACC AAA TTC	58	NC_000014.9	18/271 bp
*THRB*	F: GTG TTA TTA GTT TGA TTA TTT GTT R: CTA TTC CAC CAC TAT CCA C	54	NC_000003.12	11/128 bp

Ta, appropriate annealing temperature; CpG, cytosine–phosphate diester–guanine

Human methylated and unmethylated DNA sets from Zymo Research were used as 100% methylated and 0% unmethylated controls. Series of standard dilutions of methylated DNA were prepared. For *THRB* gene, methylation percentages of 0, 1, 5, 10, 25, 50, and 100% were used to draw the standard curve, while standards of 0, 50, 60, 70, 90, and 100% were used for *DIO3*. Melting curves were normalized relative to two normalization regions before and after a major decrease in fluorescence. This indicated the melting region of the PCR product using the HRM version 2.2 software (ThermoFisher Scientific).

### RNA extraction, cDNA synthesis, and quantitative real-time polymerase chain reaction

Total RNA was isolated from colon tissues using RNX-Plus solution according to the manufacturer's protocol (Cinaclone, Iran). Total RNA (1 μg) was used for cDNA synthesis (Cinaclone, Iran), according to the manufacturer’s instructions. The polymerase chain reaction (PCR) for *THRB*, *DIO3*, and glyceraldehyde-3-phosphate dehydrogenase (*GAPDH*) (as an internal control) was performed in duplicate by 10 μL BIOFACT™ 2X real-time PCR master mix (for SYBR Green I; High Rox, BIOFACT, South Korea), 7 μL double-distilled water, 0.5 μL forward primer (10 pmol/µL), 0.5 μL reverse primer (10 pmol/µL), and 2 μL cDNA in a final volume of 20 μL. After an initial denaturation step of 15 min at 95 °C, 50 cycles of amplification were carried out. Each two step cycle included a denaturation step, 15 s at 95 °C and an annealing step, 25 s at 55 °C. The melt curve was between 60 and 95 °C (StepOnePlus; Real-Time PCR, Applied Biosciences, Paisley, UK). The gene expression values were calculated as the fold change defined by 2^−ΔΔct^. The applied primers are listed in Table 2.

**Table 2:** Oligonucleotide primers used for real-time PCR analysis

Product length (bp)	Sequence (5′ → 3′)	Gene name
116	F: CGC TGG TTC TAA AGT TCG R: GAT GTA GAT GAT GAG GAA GTT G	DIO3
128	F: CAA AGT CAG GCG AAA TCA G R: CCC AGT TCT CCT CTA TCA G	THRB
120	F: CAT CAA GAA GGT GGT GAA GCA G R: GCG TCA AAG GTG GAG GAG TG	GAPDH

Bp, base pair

### Statistical analyses

Statistical tests were performed using SPSS software (version 20), and a two-sided *P* -value < 0.05 was considered significant. Independent-samples t-test was used to check differences in the distribution of continuous variables between two groups. Chi-square (χ2) test and Fisher's exact or Fisher–Freeman–Halton test were applied for categorical variables. Regression analyses were performed to assess the relationship between variables and to control confounders. As suggested elsewhere [15] confounders with univariate regression *P* value < 0.2 were included in an adjusted model. Thus, among demographic and lifestyle factors of the study subjects, only those which met the above criteria were considered as confounders.

Accordingly, relationships of DIO3 methylation levels, *DIO3* delta Ct, and *THRB* delta Ct with BMI, weight and central obesity of participants were adjusted for omega 3 fatty acid intake, red meat consumption and smoking, NSAID use, consumption of vitamin D and colon cancer family history, respectively.

## Results

### Characteristics of cases and controls

The demographic and lifestyle characteristics of participants are demonstrated in Table 3. The mean (SD) age of participants was 42.9 ± 9.5 and 38.4 ± 10.1 years in cases and controls, respectively (*P* = 0.168). Weight, BMI, WC, hip circumference, and WHR were significantly different between the two groups (*P* < 0·001). However, there was no significant difference in other characteristics.

**Table 3:** Baseline characteristics of cases and controls

Variables	Controls (n = 21)		Cases (n = 18)		*P* value*
	Mean	SD	Mean	SD	
Weight (kg)	73.14	6.22	99.1	9.1	< 0.001
Height (cm)	176.76	4.93	176.75	6.34	0.995
BMI (kg/m^2^)	23.4	1.52	31.65	1.65	< 0.001
Waist (cm)	91.0	5.35	110.92	5.64	< 0.001
Hip (cm)	100.0	3.27	113.94	5.09	< 0.001
WHR	0.91	0.04	0.97	0.05	< 0.001
Age (years)	38.4	10.1	42.9	9.5	0.168

	n (%)		n (%)		*P* value*
Cancer family history (yes)	5 (23.8)		9 (50)		0.089
Colon cancer family history (yes)	2 (9.5)		5 (27.8)		0.215
NSAID use (yes)	9 (42.9)		12 (66.7)		0.137
*Education*					0.382
Illiterate	0 (0)		0 (0)		
Under high school diploma	4 (19)		2 (11.1)		
Between the high school diploma and bachelor's degree	8 (38.1)		11 (61.1)		
Above the bachelor’s degree	9 (42.9)		5 (27.8)		
*Smoking status*					0.399
Never smoking	11 (52.4)		7 (38.9)		
Smoker	10 (47.6)		11 (61.1)		
Consumption of calcium supplement (yes)	4 (19)		1 (5.6)		0.349
Consumption of vitamin D supplement (yes)	8 (38.1)		6 (33.3)		0.757
Consumption of vitamin B supplement (yes)	9 (42.9)		5 (27.8)		0.328
Consumption of Omega 3 fatty acids supplement (yes)	3 (14.3)		2 (11.1)		1
Alcohol use (yes)	7 (33.3)		6 (33.3)		1
*Red meat consumption*					0.846
< 2/week	9 (42.9)		7 (38.9)		
2–3/week	5 (23.8)		6 (33.3)		
> 3/week	7 (33.3)		5 (27.8)		

WHR, waist to hip ratio; BMI, body mass index; NSAID, non-steroidal anti-inflammatory drugs

*Independent-samples t-test was used for continuous variables. Chi-square test and Fisher's exact or Fisher–Freeman–Halton test were used for categorical variables

### Promoter methylation status of *THRB*

**Fig. 1:**
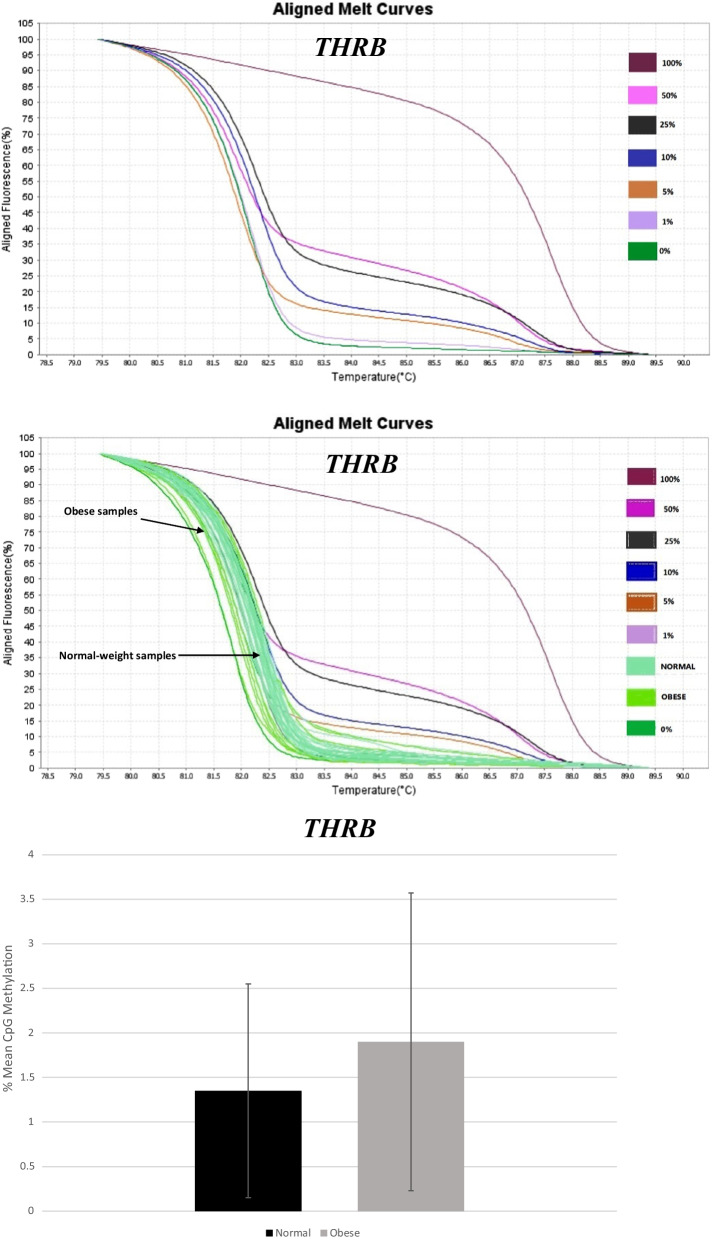
Graphs and diagram related to MS-HRM analysis of *THRB* gene promoter methylation. Aligned melt curves for *THRB* controls (**a**) and normal-weight compared to obese samples (**b**). No significant difference in mean ± SD methylation levels at *THRB* promoter region between normal-weight compared to obese samples was observed (**c**)

MS-HRM aligned melt curves analysis for *THRB* controls is shown in Fig. 1a. Figure 1b and c illustrate *THRB* promoter methylation of normal-weight group compared to obese participants, in colon sample tissues. Results indicated no significant difference in mean methylation levels at *THRB* promoter region between obese cases (1.9 ± 1.67) and normal weight controls (1.35 ± 1.20) (Fig. 1c).

There was no significant relation between methylation levels of *THRB* and demographic/lifestyle factors of the participants. There were also no significant relations between *THRB* methylation levels and BMI, weight or central obesity, in both crude and adjusted models.

### Promoter methylation status of *DIO3*

**Fig. 2:**
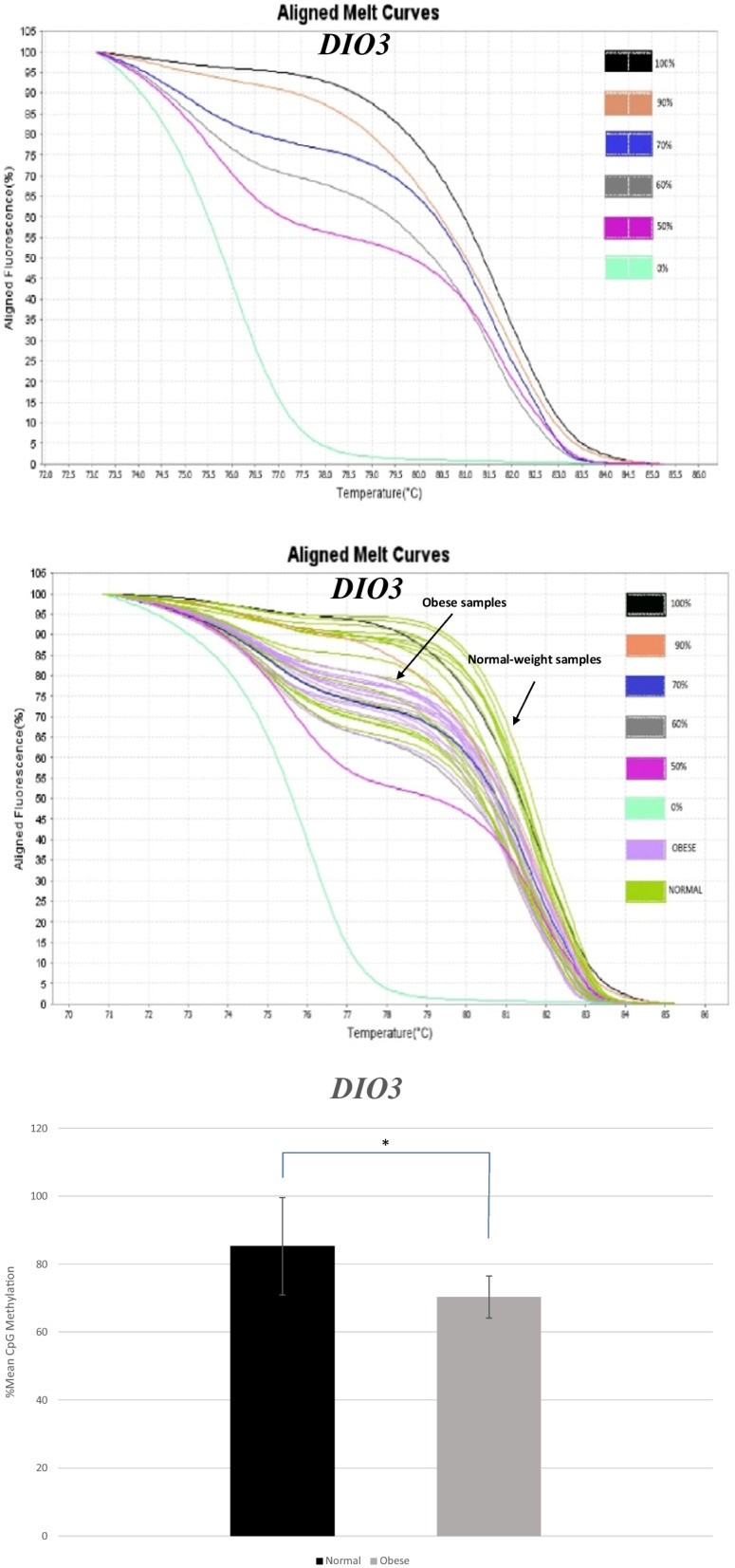
Graphs and diagram related to MS-HRM analysis of *DIO3* gene promoter methylation. Aligned melt curves for *DIO3* controls (**a**) and normal-weight compared to obese samples (**b**). There was a significant difference in mean ± SD methylation levels at *DIO3* promoter region between obese and normal-weight samples. **c** *P < 0.001 between normal-weight and obese subjects

MS-HRM aligned melt curves analysis for *DIO3* controls and normal-weight compared to obese samples are presented in Fig. 2a and b. MS-HRM analysis of *DIO3* showed that there was a significant difference in mean methylation levels at the *DIO3* promoter region between obese cases (70.32 ± 6.16) and normal-weight controls (85.26 ± 14.34) (*P* < 0.001) (Fig. 2c).

As shown in Table 4, simple linear regression was used to examine the relation between *DIO3* methylation levels and demographic/lifestyle factors of the study participants. Among demographic/lifestyle factors only omega-3 fatty acid intake showed a significant direct association with *DIO3* methylation levels (*P* < 0.05).

**Table 4:** Relationship between methylation levels and demographic/lifestyle factors of the study participants in *DIO3* gene

Characteristics	*DIO3* methylation%	
	β^A^	*P* value*
Age	− 0.145	0.384
Consumption of vitamin B supplement	0.027	0.872
Consumption of calcium supplement	0.003	0.987
Consumption of vitamin D supplement	− 0.085	0.611
NSAID use	− 0.123	0.460
Consumption of Omega 3 fatty acids supplement	0.399	0.013
Colon Cancer family history	0.128	0.442
Cancer family history	0.142	0.395
Alcohol consumption	− 0.062	0.712
*Red meat consumption*		
< 2/week	Reference	
2–3/week	0.293	0.231
> 3/week	0.300	0.220
*Smoking*		
Never smoking	Reference	
Smoker	− 0.192	0.248

NSAID, Non-steroidal Anti-inflammatory Drugs

*Simple linear regression was used to study the linear relationship between variables

^A^β refers to the standardized coefficient

The relations between *DIO3* methylation levels and BMI, weight, and central obesity of cases are demonstrated in Table 5. A significant inverse relationship was observed between BMI and methylation levels of *DIO3* in crude (*P* < 0.001) and adjusted (*P* < 0.001) analyses. A significant inverse relation was also seen between crude and adjusted analyses of central obesity and methylation levels (*P* < 0.05). As for weight, the crude model showed a significant inverse relation (*P* < 0.05) while, the adjusted model showed an approximately significant inverse relation with *DIO3* methylation levels (*P* = 0.057).

**Table 5:** Relationships between *DIO3* methylation levels and BMI, weight and central obesity of study participants

	*DIO3* Methylation%	
	β^B^	*P* value^A^
*BMI*		
Crude	− 0.608	< 0.001
Adjusted	− 0.534	< 0.001
*Weight*		
Crude	− 0.387	0.016
Adjusted	− 0.301	0.057
*Central obesity*		
Crude	− 0.470	0.003
Adjusted	− 0.376	0.019

Multiple linear regression and simple linear regression were used to study the linear relationship between variables

^A^Adjusted for omega 3 fatty acid consumption

^B^β refers to the standardized coefficient

−Confounders with *P* < 0.2 included in the adjusted analysis

### Analyses of *THRB* and *DIO3* gene expressions in case and control groups

**Fig. 3:**
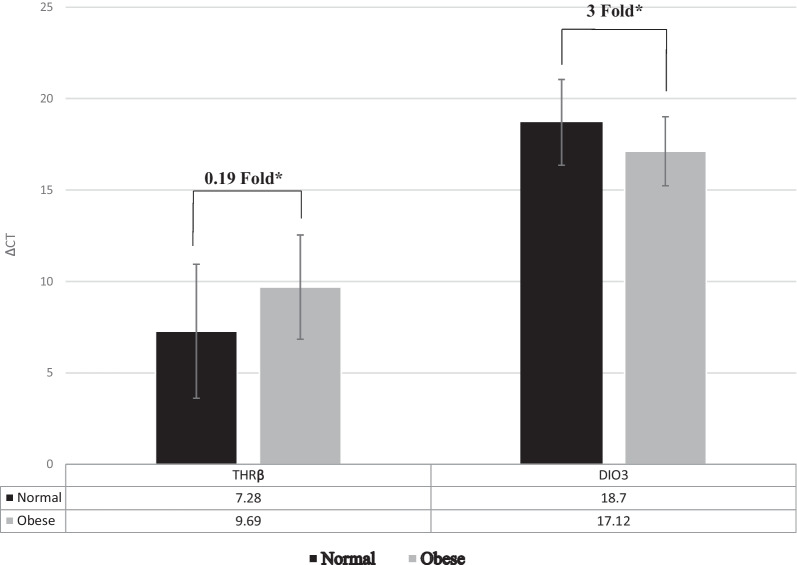
ΔCT mean ± SD values of *DIO3* and *THRB* gene expressions between normal-weight and obese participants. There was a significant difference in ΔCT mean values of *THRB* between obese (9.69 ± 2.85) and normal-weight samples (7.28 ± 3.66). A significant difference in ΔCT mean values of *DIO3* between obese (17.12 ± 1.89) and normal-weight samples (18.7 ± 2.35) was observed. Gene expression results were expressed as the fold change defined by 2^– ΔΔct^. The higher ΔCT values indicate lower gene expression. *P < 0.05 between normal-weight and obese subjects

After analysis of real-time PCR raw data and analysis of expression fold change, our results indicated that the level of *THRB* gene expression was significantly lower in obese group compared to controls (*P* < 0.05; fold change: 0.19). The level of *DIO3* gene expression in obese cases was significantly higher than in controls (*P* < 0.05; fold change: 3) (Fig. 3).

There was no significant association between delta Ct of *THRB* and demographic/lifestyle factors. In addition, no significant relation was observed between delta Ct of *DIO3* and demographic/lifestyle factors.

The relations of *THRB* and *DIO3* delta Ct with BMI, weight, and central obesity of the study subjects are demonstrated in Table 6. There was a direct significant relation between *THRB* delta Ct and BMI in the crude analysis (*P* < 0.05). A significant direct relation was observed between *THRB* delta Ct and weight in the crude analysis (*P* < 0.05). Nevertheless, for the adjusted analysis, the relation of *THRB* delta Ct with BMI and its relation to weight were marginally significant, (*P* = 0.054 and *P* = 0.056, respectively). Moreover, *THRB* delta Ct relation with central obesity was not significant.

**Table 6:** Relationships of *DIO3* and *THRB* ΔCT with BMI, weight and central obesity of study participants*

	*DIO3* ΔCT		*THRB* ΔCT	
	β^C^	*P* value^A^	β^C^	*P* value^B^
*BMI*				
Crude	− 0.365	0.022	0.382	0.017
Adjusted	− 0.315	0.050	0.302	0.054
*Weight*				
Crude	− 0.323	0.045	0.371	0.020
Adjusted	− 0.300	0.061	0.307	0.056
*Central obesity*				
Crude	− 0.300	0.063	0.260	0.110
Adjusted	− 0.247	0.143	0.213	0.200

Multiple linear regression and simple linear regression were used to study the linear relationship between variables

^A^Adjusted for red meat consumption and smoking

^B^Adjusted for NSAID use, consumption of vitamin D and colon cancer family history

^C^β refers to the standardized coefficient

−Confounders with *P* < 0.2 included in the adjusted analysis

A statistically significant inverse relation was observed between *DIO3* delta Ct and BMI in the crude model (*P* < 0.05. A perceivable statistically significant inverse association was seen in the adjusted model (*P* = 0.05). A significant inverse relation was also observed between *DIO3* delta Ct and weight in the crude analysis (*P* < 0.05). The adjusted model approached near significance (*P* = 0.061). Moreover, the inverse relation of *DIO3* delta Ct with central obesity approached statistical significance in the crude analysis (*P* = 0.063), and was not significant in the adjusted model.

## Discussion

The present study showed that *THRB* is downregulated in epithelial colon tissues of obese individuals. Moreover, obesity induces hypomethylation of *DIO3* promoter and its upregulation, consistently. Obesity has a possible influence on epigenetic landscape that is related to CRC [16]. Moreover, the results of other studies also showed that DNA methylation changes in obesity-related CRC, can be considered as a marker for predicting CRC [17, 18]. A study showed that alterations of DNA methylation and gene expression induced by obesity in the colonic epithelium of a mouse model, promoted pro-proliferative signaling pathways, but, weight loss reversed these changes [19].

A little increase in promoter methylation and low expression of *THRB*, in obese individuals, may arise from the unequal link between gene expression and methylation. [20]. Based on a study, liver *THRB* expression negatively correlated with a severe nonalcoholic steatohepatitis in obese individuals who had undergone bariatric surgery [21]. In another study, *THRB* gene expression was significantly lower in adipose tissues of obese patients compared to normal-weight counterparts [22]. Furthermore, Zhu et al. (2016) revealed that *THRB1* gene expression was reduced in CRC tumors compared to normal colorectal mucosal tissues [11]. Even though obesity predisposes individuals to oncogenic events, some studies have reported that p53 and even p16 tumor suppressors became activated in some tissues of obese individuals [23–25]. Although the role of THs in cancer is uncertain, several pioneering studies have identified *THRB1* as a tumor suppressor gene in different cancers, particularly in CRC [11]. A possible suggested mechanism is that *THRB1* in colon cells can stimulate degradation of β-catenin, thus, inactivating Wnt signaling pathway [26]. *THRB1* expression can also suppress progression and migration by preventing PI3K/Akt signaling in CRC tissues and cells [11]. An innovative study revealed that TRβ1 was highly expressed in differentiated cells of the surface and upper colon crypt, compared to cells with high stemness located in lower crypt. However, *THRA* expression showed an opposite manner [27].

Our study showed that obesity attenuated DNA methylation and accordingly enhanced the expression of *DIO3*. Although there is a lack of research on *DIO3* gene methylation and expression in obesity, one research showed that *DIO3* mRNA was increased in omental and subcutaneous white adipose tissues of obese men and women patients compared to their lean counterparts [28]. The human oncofetal *DIO3* is frequently re-expressed in some conditions, including chronic inflammation, critical illness, starvation and some cancers. Furthermore, signaling pathways linked to stemness such as sonic hedgehog-glioma associated oncogene 2 (Shh-Gli2), tumor growth factor-β (TGF-β), Wnt/β-catenin, and hypoxia-inducible factor-1α (HIF-1α) increase the activity of the *DIO3* enzyme [29]. Furthermore, obesity as an inflammatory disease, might trigger inflammation in the colon, as well. It seems that inflammation upregulates Wnt/β-catenin through NF-κB (nuclear factor kappa-light-chain-enhancer of activated B cells) [30]. *DIO3* is a direct β-catenin/TCF complex target [13]. In addition, it has been indicated that local inflammation can greatly enhance *DIO3* activation in inflammatory cells, particularly in invading polymorphonuclear granulocytes [31, 32]. Therefore, up and down-regulation of *DIO3* and *DIO2*, by over-activation of the Wnt/β-catenin pathway, in colon carcinomas, are significant contributors to colorectal carcinogenesis [13, 33]. Consequently, based on our findings, high expression of *DIO3* gene in obese individuals might be a factor that predisposes tissues to neoplastic transformation.

Mechanisms of resistance to TH (RTH) have been updated. Mutations in TRs are not the only condition that reduces activity and availability of TH in target tissues. Defects in the synthesis of deiodinases may also exist in RTH [34, 35]. Moreover, RTH is connected to etiology of obesity where changes occur in the expression and activity of deiodinases and TRs [36, 37]. Based on our results, altered thyroid hormone bioavailability may have happened in epithelial colon tissues of obese adults.

Among nutritional and dietary factors presented here, only omega-3 fatty acid consumption was directly related to increasing methylation levels of the *DIO3* promoter gene. This finding is in agreement with studies that have reported the role of n-3 and n-6 PUFA on DNA methylation status [38]. By considering factors missed in our study, such as physical activity, stress, environmental and metabolic factors, further research is needed to confirm the exact role of omega-3 fatty acid in preventing colorectal cancer.

## Conclusions

Obesity dysregulates epigenetics and expression of *DIO3,* and expression of *THRB*. It may also alter thyroid hormone bioavailability in human colon tissues. These changes induced by obesity could eventually result in CRC. Since this study provided no evidence to support CRC development in the participants, and current literature regarding clinical significance of TRβ and DIO3 at protein levels is inadequate, more evidence on obese individuals is required for further therapeutic approaches.

## Data Availability

The data that support the findings of this study are openly available in 4TU.ResearchData at https://doi.org/10.4121/14789856.v1, reference number [39].

## Abbreviations

CRC: Colorectal cancer
THRB: Thyroid hormone receptor beta gene
DIO3: Type 3 deiodinase
MS-HRM: Methylation sensitive-high resolution melting
qRT-PCR: Quantitative real-time polymerase chain reaction
THs: Thyroid hormones
TRs: Thyroid hormone receptors
TRα: Thyroid hormone receptor α protein
TRβ: Thyroid hormone receptor β protein
PI3K: Phosphoinositide 3-kinase
MAPK: Mitogen-activated protein kinase
TCF: β-Catenin/T-cell factor
BMI: Body mass index
WHR: Waist:hip ratio
WC: Waist circumference
PCR: Polymerase chain reaction
GAPDH: Glyceraldehyde-3-phosphate dehydrogenase
Shh-Gli2: Sonic hedgehog-glioma associated oncogene 2
TGF-β: Tumor growth factor-β
HIF-1α: Hypoxia-inducible factor-1α
NF-κB: Nuclear factor kappa-light-chain-enhancer of activated B cells
RTH: Resistance to thyroid hormone
